# Bioresorbable flow-diverters for cerebral aneurysms: a systematic review

**DOI:** 10.1007/s00234-026-03908-x

**Published:** 2026-02-03

**Authors:** Matteo Palermo, Andrea Alexandre, Alessio Albanese, Alessandro Pedicelli, Mostafa Abdulrahim, Alessandro Olivi, Carmelo Lucio Sturiale

**Affiliations:** 1 Department of Neurosurgery, Fondazione Policlinico Universitario A. Gemelli IRCCS, Università Cattolica del Sacro Cuore, Rome, Italy; 2 Department of Neurosurgery, Khatib Brain Tumor Center, Johns Hopkins University School of Medicine, Baltimore, MD USA

**Keywords:** Bioresorbable, Flow diverter, Stent, Aneurysm, Brain, Cerebral

## Abstract

**Background:**

Bioresorbable flow diverters (BRFDs) are emerging as next-generation alternatives to permanent metallic stents for intracranial aneurysm flow diversion. These devices aim to provide temporary flow diversion that scaffolds aneurysm healing and then dissolves, potentially reducing chronic foreign-body risks.

**Methods:**

We performed a systematic review (PRISMA methodology) updated to August 2025 to assess BRFD technology and performance. Seventeen studies (2013–2025) met inclusion criteria, describing 27 prototype BRFDs.

**Results:**

Devices varied widely in material and design. Many were fully polymeric (typically poly–L-lactic acid or polycaprolactone) or used biodegradable metals (FeMnN), often in polymer–metal hybrid configurations. Approximately 30% were deliverable via standard microcatheters, while about 30% required balloon expansion for wall apposition. In vivo aneurysm models, showed that 61% of BRFDs induced ≥ 80% aneurysm sac occlusion and 33% achieved complete occlusion by final follow-up. These occlusions typically appeared by 3–6 months post-implantation. No parent or jailed branch vessel occlusions were reported. About half of devices fully degraded by study end, and even after polymer or magnesium resorption any integrated radiopaque markers remained in place; importantly, no significant chronic inflammation or vessel stenosis was observed post-resorption.

**Conclusion:**

No BRFD has yet entered clinical practice. Conclusions regarding efficacy, safety, and long-term durability remain theoretical, and that the persistence of aneurysm occlusion after complete scaffold resorption has not yet been established.

**Supplementary Information:**

The online version contains supplementary material available at 10.1007/s00234-026-03908-x.

## Introduction

Intracranial aneurysms (IAs) are pathological dilations of cerebral arteries that carry the risk of life-threatening rupture. In particular, large or giant untreated aneurysms can have a 5-year rupture risk as high as 15–50%, depending on specific aneurysm characteristics such as size, neck width, morphology, and location, and rupture often leads to subarachnoid hemorrhage with considerable morbidity and mortality [1]. To prevent rupture, the only widely adopted treatments for many years were surgical clipping of the aneurysm neck and endovascular coiling of the aneurysm sac [1]. In the last two decades, flow diversion emerged as a transformative endovascular strategy for difficult aneurysms. Flow-diverting stents are high-density mesh implants deployed in the parent artery across the aneurysm neck, redirecting blood flow away from the sac [2, 3]. By attenuating intra-aneurysmal flow, these devices induce thrombosis within the aneurysm and serve as a scaffold for endothelialization across the neck [1]. This parent vessel remodeling technique has enabled curative treatment of many large or wide-neck IAs that were previously untreatable by coils alone [3].

All currently approved flow-diverters are made of permanent metal alloys that remain in the patient for life (Table 1) [1, 4–7]. The implanted stent is a foreign body that can trigger chronic arterial inflammation and neointimal hyperplasia, sometimes leading to in-stent stenosis or late-onset thrombosis occurring well after device implantation [9, 10]. To mitigate acute thromboembolism, patients must undergo prolonged antithrombotic therapy, typically dual antiplatelet therapy (DAPT) for several months, after FD placement [1, 9, 11, 12]. This requirement for long-term medication increases bleeding risk and can be problematic for patients with contraindications to antiplatelets. Moreover, the permanent metal scaffold may pose challenges down the line: it can obstruct small arterial branches covered by the stent, hinder normal vasoreactivity of the vessel, and produce artifacts on imaging that obscure adjacent brain structures [13]. In young patients, a lifelong metal implant is especially concerning, not because of significant ongoing cerebrovascular growth after early childhood, but due to its permanent long-term biological interaction with the vessel wall and the lack of reversibility if the device is no longer needed over decades of life.

**Table 1:** Features of FDA-approved flow diverters

Device	Company	Primary composition	Strut thickness (microm)	Radio-opaque marker	Porosity (%)	Pore density (pores/mm^3)	Device dimensions (diameter x length, mm)	Baloon required	Microcatheter compatible
Pipeline embolization device [4–8]	Medtronic	36 cobalt chromium wires	28–33	12 Platinum- tungsten wires	65–70	13–14	2.5–5.5 × 10–35	No	Yes
Surpass evolve [5]	Stryker	52 cobalt chromium wires	25–32	12 Platinum- tungsten wires	70	15–30	2–5 × 12 × 50	No	Yes
FRED [4, 5]	Microvention	48 wire inner layer, 16 wire outer layer, nitinol	23–36 (inner) 51–56 (outer)	Tantaluum markers	50–60	20	2.5–5.5 × 12–45	No	Yes

Bioresorbable flow diverters (BFDs) have been proposed as an innovative solution to overcome all the drawbacks related to classical implants [13].Clinical studies of bioresorbable coronary stents have also shown a return in vasomotor function following device resorption [13]. Given the potential advantages, researchers are now exploring BFDs as the next-generation flow diversion technology [1].

The rapid technological advances and the large number of studies reported in recent years, particularly those addressing the newest second-generation BRFDs, made of new alloys and composite materials, which were not yet available at the time of the only prior systematic review by Oliver et al., make an updated synthesis of the literature both timely and necessary [13]. This review was conducted with the explicit understanding that the reported findings are preclinical in nature and that clinical translation has yet to be established.

## Methods

**Fig. 1:**
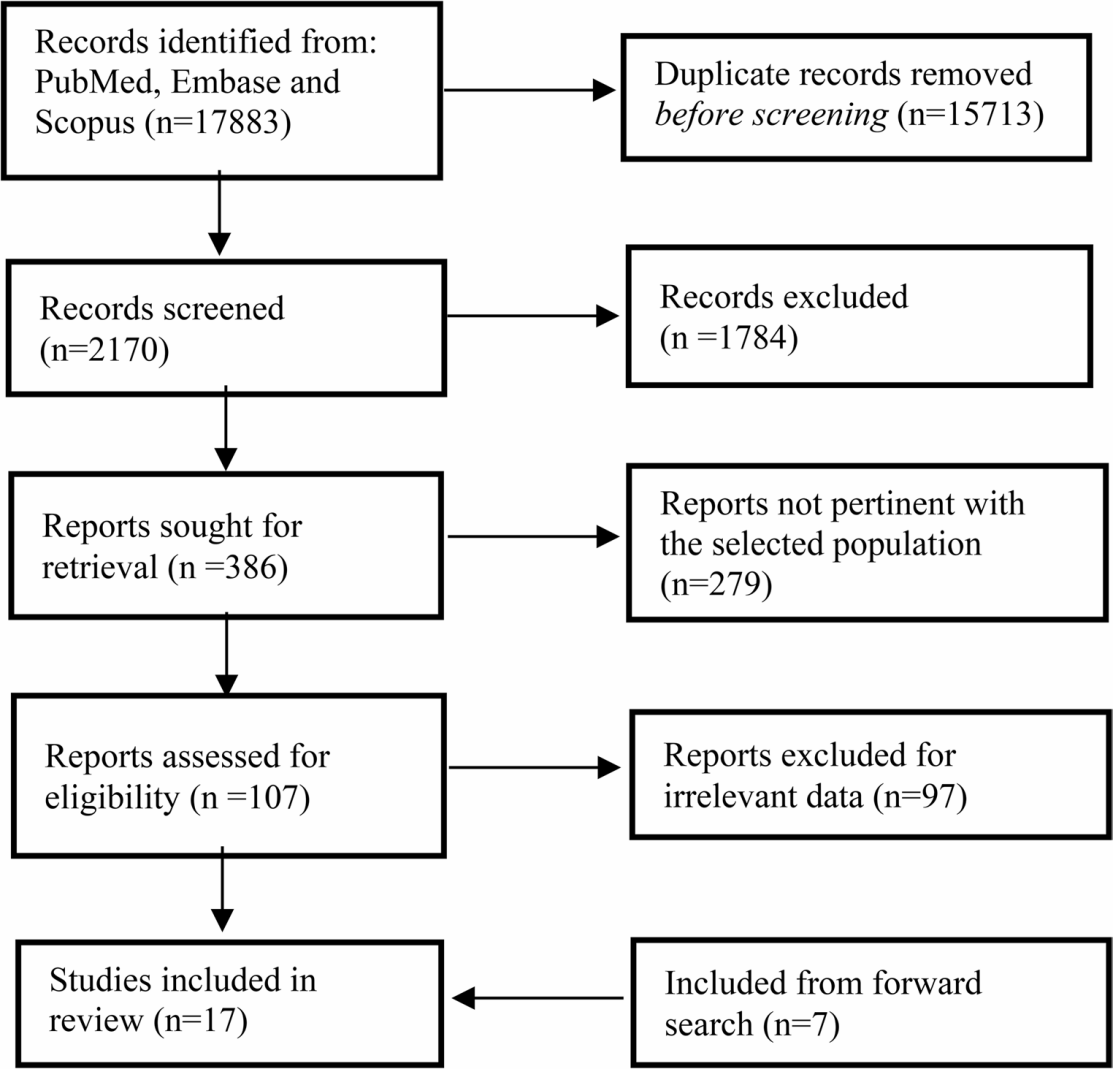
PRISMA flowchart of study selection process

This review was performed according to the PRISMA (Preferred Reporting Items for Systematic Reviews and Meta-Analyses) 2020 guidelines [14]. The PEO framework (Population: preclinical models; Exposure: bioresorbable flow diverters; Outcomes: occlusion, biological response, and resorption) was used to formulate the research question (Fig. 1).

### Search strategy

Two researchers (CLS and MP) carried out an extensive literature search using the PubMed/MEDLINE, Scopus, and EMBASE databases to find clinical, in vivo, and in vitro studies focused on the use of bioresorbable flow-diverters for treating IAs. The search strategy used is the following: *((bioresorbable OR bio-resorbable OR resorbable OR bioabsorbable OR bio-absorbable OR absorbable OR biodegradable OR bio-degradable OR biopolymer OR biomaterial OR degradable OR dissolvable) AND (flow diverter OR flow-diverter OR flow diverting OR flow-diverting OR flow diversion OR flow-diversion OR stent OR stents OR stenting)).* The search was restricted to studies published in English and was last updated on August 14, 2025. To ensure a comprehensive review, the authors also performed a forward citation search of the reference lists from all included articles.

### Study selection

The search was restricted to peer-reviewed, English-language studies. We included studies that reported use of bioresorbable devices for the treatment of IAs. For the purposes of this study, pre-clinical studies were also selected. We excluded cases in which bioresorbable stents were used solely for coil-assisted embolization or in which non-BRFDs were employed, as the aim of this review was to assess BRFDs for intracranial aneurysms. Reports involving bioresorbable vascular scaffolds or stents designed for applications outside the intracranial circulation, like coronary or peripheral arteries, preclinical models unrelated to aneurysm treatment, or hybrid constructs in which the flow-diversion component was non-bioresorbable were also excluded. Review articles and studies lacking sufficient clinical or diagnostic details were excluded.

Two authors (CLS and MP) independently reviewed the titles and abstracts of all articles identified through the search process and selected studies based on the pre-established inclusion and exclusion criteria. Any discrepancies between their selections were addressed through discussion until they reached a consensus.

After removing ineligible articles, the full texts of the remaining studies were carefully examined to confirm eligibility, following the same criteria (Fig. 1). In cases of disagreement, a consensus meeting was held where the article and extracted data were re-assessed (BLINDED) by two experienced neuroradiologists, to resolve any differences.

### Data extraction

For each eligible study, we extracted the author and year of publication. Subsequently, we identified experimental bioresorbable flow-diverters described in the literature (Table 2). For each device, we recorded the manufacturer and primary composition, as well as key biomechanical characteristics such as strut thickness, radio-opaque markers, porosity, pore density, and device dimensions. We also assessed whether the devices were compatible with existing microcatheters and whether balloon angioplasty was required to achieve adequate wall apposition and aneurysm sealing.

**Table 2:** Features of the experimental flow diverters reviewed

Author, year	Device (Gener.)	Device material	Strut thickness (µm)	Radio-opaque marker	Porosity (%)	Pore density (pores/mm^2^)	Braid angle (degrees)	Dimension (diameter x length, mm)	Fully bioresorbable	Microcatheter compatible (inner diameter)	Balloon required	Deployment notes
Wang et al. 2013 [15]	BRFD 1st	22 nitinol wires, 24 polyglycolic acid wires	45*	Two parallel platinum containing struts	50–60	12–20	40*	3.5 and 4.5 mm diameters, length not provided	No	Yes (0.027″)	No	The FD was advanced through a compatible microcatheter and deployed across the aneurysm neck. Device sizing was chosen such that the open device diameter exceeded the parent artery diameter by ≤ 1 mm.
Wang et al. 2013 [15]	BRFD 1st	22 nitinol wires	25–30*	Two parallel platinum- containing struts	80–85	3–5	45	3.5 and 4.5 mm diameters, length not provided	Fully permanent	Yes	No	
Nishi et al. 2019 [11]	BRFD 1st	48 poly- L- lactic acid wires	40–45	Three radio- opaque gold markers at both device ends	40*	NA	40*	4 × 10, 4 × 12, and 4 × 15	Yes	Yes	Yes	Transfemoral approach. Push–pull deployment across the aneurysm neck. Self-expanding braided scaffold. Routine post-deployment balloon angioplasty to ensure full wall apposition
Jamshidi et al. 2020 [9]	BRFD 1st	44/4. 46/2, and 48/0 poly- L- lactic acid/tantalum coated nitinol wires	50	Tantalum coated nitinol wires	60	17	50*	4 × 10–20	No	Yes (0.027″)	No	Self-expanding. Delivered through a microcatheter. No adjunctive angioplasty required. Achieved good wall apposition. No migration observed
Tidwell et al. 2021 [12]	BRFD 1st	Polycaprolactone	350	BaSO4 coating	65	0.87	NA	5.5 × 15	Yes	NA	NA	NA
Gruter et al. 2019 [16]	BRFD 1st	Magnesium alloy coated in poly- L- lactic acid	120	None	Not available (NA)	NA	NA	2.5 × 6	Yes	NA	Yes	NA
Catalán-Echeverría et al. 2019 [17]	BRFD 2nd	Polymer (DBRP)	400	NA	90*	NA	20*	4.75 × 40	Yes	NA	No	NA
Rezabeigi et al. 2022 [18]	BRFD 2nd	ReSolv: Hybrid: ≥40 PLLA polymer fibers + up to 8 platinum-based radiopaque strands (braided)	40–50*	Radiopacity from integrated platinum strands	60–65*	18–25*	65–75*	NA	No	NA	No	NA
Muram et al. 2022 [19]	BRFD 2nd	48-filament: ≥40 bioresorbable polymer fibers + ≤ 8 metal strands	Not available (NA)	Metal strands are radiopaque + marker bands at each end	58.5–71.2	8.8–21.2	NA	4.5 × 36	No	Yes	No	Deployment was performed in a rabbit elastase-induced saccular aneurysm model via femoral access using a 6 F sheath. A microcatheter was advanced through a guide catheter, and the device was deployed using a push–pull technique. No balloon angioplasty was required. Immediate angiographic assessment was performed using the O’Kelly–Marotta (OKM) scale and the 4 F flow-diversion predictive score (4 F-FPS).
Morrish et al. 2022 [20]	BRFD 2nd	Braided self-expanding polymer/metal hybrid: poly-L-lactic acid (PLLA) + radio-opaque composite wire	25–55*	Terminal bands at each end	60–75*	15–20*	62–70*	4.0 × 22	No	NA	NA	NA
Belanger et al. 2024 [8]	BRFD 2nd (unsh)	ReSolv: Hybrid metal/polymer (PLLA)	60–120*	NA	80*	NA	60*	3.5 × 20	No	NA	NA	NA
Belanger et al. 2024 [8]	BRFD 2nd (sh)	ReSolv: Hybrid metal/polymer (PLLA)	60–120*	NA	80*	NA	60–120*	3.5 × 20	No	NA	NA	NA
Sasaki et al. 2023 [21]	BRFD 2nd	Poly(L-lactic acid), braided (48 fibers)	41.7	3 gold markers 1 mm inside each end	60	20	NR	4 × 10–15	Yes	NR	No	Devices were deployed to cover the aneurysm neck (proximal to the vertebral artery origin) and a lumbar artery ostium in the abdominal aorta. Balloon angioplasty was not used with the PLLA-FD. Balloon angioplasty was performed only in the CoCr-FD group when malapposition was suspected. Wall apposition was systematically assessed with OCT.
Sasaki et al. 2023 [21]	BRFD 2nd	36 cobalt-chromium + 12 platinum-tungsten wires	30	NR	68	21	NR	4 × 10–15	No	NR	No	
Hossan et al. 2023 [22]	BRFD 2nd	Polycaprolactone	74	BaSO₄ or Bi₂O₃ coating	50	8.45	NA	3 × 10	Yes	Yes	No	NA
Hossan et al. 2023 [22]	BRFD 2nd	Polycaprolactone	63	BaSO₄ or Bi₂O₃ coating	60	9.26	NA	3 × 10	Yes	Yes	No	NA
Hossan et al. 2023 [22]	BRFD 2nd	Polycaprolactone	57	BaSO₄ or Bi₂O₃ coating	70	9.85	NA	3 × 10	Yes	Yes	No	NA
Oliver et al. 2023 [23]	BRFD 2nd	WE22 Mg alloy (> 95% Mg + rare earths) + 8 Ta wires (polyimide-coated)	Mg 50; Ta 30 (40 coated)	Tantalum (8 wires), polyimide-coated	81	4.3	64	4.75 × 15	No	NA	No	NA
Oliver et al. 2023 (1) [23]	BRFD 2nd	FeMnN alloy (Fe + 35% Mn + 0.15% N) + 12 Ta wires (polyimide-coated)	Fe 25; Ta 30 (40 coated)	Tantalum (12 wires), polyimide-coated	79	9.9	64	4.75 × 15	No	NA	No	NA
Oliver et al. 2023 (2) [24]	BRFD 2nd	Braided magnesium alloy wires with tantalum marker wires	Mg wires: 50; Ta wires: 30	Tantalum wires integrated in braid	72–85*	NR	NR	4.75 × 10	No	NA	NA	NA
Oliver et al. 2023 (2) [24]	BRFD 2nd	Braided antiferromagnetic iron alloy wires with tantalum marker wires	Fe wires: 25; Ta wires: 30	Tantalum wires integrated in braid	75–85*	NR	NR	4.75 × 10	No	NA	NA	NA
Akiyama et al. 2024 [25]	BRFD 2nd	Magnesium alloy (KUMADAI Mg; braided 48 wires)	46	Gold markers, 3 per end	64	16	NA	4.0 x diameters, length not provided	Yes	NA	Yes	In vivo implantation in rabbit abdominal aorta. Femoral arterial access. Routine balloon angioplasty performed to improve wall apposition. Device evaluation using angiography, OCT, micro-CT, and SEM
Akiyama et al. 2024 [25]	BRFD 2nd	Magnesium alloy wire with 5 μm PLLA coating	56	Gold markers, 3 per end	63	12	NA	4.0 x diameters, length not provided	Yes	NA	Yes	
Morrish et al. 2024 [26]	BRFD 2nd (ReSolv)	≥ 40 poly-L-lactic acid (PLLA) fibers + up to 8 platinum-based radio-opaque strands (hybrid, primarily polymer)	Not available (NA)	Platinum-based strands (embedded)	Not available (NA)	NA	NA	NA	No	Yes	No	Deployed using a push–pull technique. Deployment tested in patient-specific aneurysm models, including bifurcation aneurysms.
Oliver et al. 2025 [27]	BRFD 2nd	36 wires Fe–Mn–N alloy (35% Mn, 0.15% N, balance Fe) + 12 permanent tantalum wires coated with polyimide	25	Tantalum	73*	25*	145*	4.75 × 7	No	NA	Yes (3/7)	Deployed using a push–pull technique through femoral access. Devices were deployed to cover the aneurysm neck. Balloon angioplasty performed to improve wall apposition.

*Indicates that the value was estimated using ImageJ to take measurements on published figures. *BRFD* Bioresorbable flow diverter; *NA* Not available, the metric exists for the device but figures that could be used to calculate the metric were not provided in the original manuscript; *FD* flow diverter; NA, the given metric cannot be measured for the device; (sh), shelved; (unsh), unshelved

Additional parameters including the braid angle and the device biosorption capability were also recorded.

Finally, we summarized the findings from in vivo experimental studies involving bioresorbable flow-diverters (Table 3). In these cases, we extracted details of the animal model and study protocol, duration of follow-up, and, when reported, aneurysm occlusion rates, incidence of in-stent thrombosis, and rate of device resorption.

**Table 3:** Summary of experimental results for reviewed BRFDs investigated in vivo

Author, year	Device	Study model	Study duration (sample size)	Aneurysm occlusion rates	In device thrombosis	Device resorption
Wang et al. 2013 [15]	BRFD	Rabbit elastase- induced aneurysm	6 weeks (*n* = 6) and 3 months (*n* = 7)	67% (4/6) at 6 weeks 83% (5/6) at 3 months	One case of device- induced thrombosis that resulted in the complete occlusion of the parent artery at 3 months	Residual PGA struts were observed at 6 weeks but appeared to be completely resorbed by 3 months via stereomicroscope
Wang et al. 2013 [15]	BRFD	Rabbit elastase- induced aneurysm	3 months (*n* = 7)	0% (0/6)	No observed device- induced thrombosis by gross or histological evaluation	NA
Nishi et al. 2019 [11]	BRFD	Rabbit elastase- induced aneurysm	1 (*n* = 5), 3 (*n* = 5), 6 (*n* = 5), and 12 (*n* = 3) months	0% (0/5) at 1 month, 20% (1/5) at 3 months, 50% (2/4) at 6 months, and 33% (1/3) at 1 year	No observed downstream arterial thrombus formation or occlusion via angiography. By OCT, thrombus was observed in 1.9%, 1.8%, 0.0%, and 0.0% of frames at 1, 3, 6, months and 1 year, respectively. All thrombi detected by OCT were small white thrombi	In vitro testing demonstrated 15%, 45%, 83%, and 95% reductions in weight average molecular weight at 3 months, 9 months, 1 year, and 1.5 years, respectively
Jamshidi et al. 2020 [9]	BRFD	Rabbit infrarenal abdominal aorta	1 month (*n* = 3)	NA	By SEM analysis, some thrombus was observed on the abluminal side of struts that were not opposed well to the vessel wall	No reported analysis
Gruter et al. 2019 [16]	BRFD	Rat aortic sidewall aneurysm	4 weeks (*n* = 26), serial OCT out to 6 months (*n* = 8)	85% (22/26) at 4 weeks	No reported analysis	Qualitative microCT and OCT demonstrated progressive resorption
Gruter et al. 2019 [16]	BRFD + coil	Rat aortic sidewall aneurysm	4 weeks (*n* = 7)	100% (7/7)	No reported analysis	NA
Gruter et al. 2019 [16]	Control FD + coil	Rat aortic sidewall aneurysm	4 weeks (*n* = 6)	83% (5/6)	No reported analysis	NA
Rezabeigi et al. 2022 [18]	BRFD	Rabbit elastase- induced aneurysm	18 months (*n* = 24)	NA	None	NA
Rezabeigi et al. 2022 [18]	BRFD	Rat aortic sidewall aneurysm	6 months (*n* = 6)	5/6 (83%) occlusion or near-occlusion at mean 7.5 mo (OKM)	None	NA
Muram et al. 2022 [19]	BRFD	Elastase-induced rabbit aneurysm model	4, 8, 12, 26 weeks (*n* = 29)	100% occlusion at 26 weeks (*n* = 8)	None	Progressive polymer degradation; 100% bioresorption by 1 year, metal strands remain
Morrish et al. 2022 [20]	BRFD	Swine carotid sidewall aneurysms	6 months (*n* = 3)	100% occlusion at 6 months	None	Progressive radiolucency on CT and signal loss on MRI consistent with partial polymer resorption by 6 months (markers remained visible)
Sasaki et al. 2023 [21]	BRFD	Rabbit elastase-induced aneurysm	1, 3, and 12 months (*n* = 15)	73% at 3 mo, 100% at 12 mo	No reported analysis	Progressive degradation; scanning electron microscopy showed 50% fiber mass loss by 12 mo
Sasaki et al. 2023 [21]	BRFD	Rabbit elastase-induced aneurysm	1, 3, and 12 months (*n* = 15)	80% at 3 mo, 100% at 12 mo	No reported analysis	No reabsorption noted
Morrish et al. 2024 [26]	BRFD	Rabbit elastase-induced aneurysm	1, 3, and 6 months (*n* = 18)	Complete occlusion at 6 months: 100%	None	Partial resorption of PLLA component by 6 months; platinum strands remained visible
Akiyama et al. 2024 [25]	BRFD	Rabbit elastase-induced aneurysm	1 month & 3 months (*n* = 4)	1 mo: 2/4 complete occlusion (50%); 3 mo: 4/4 complete occlusion (100%)	No reported analysis	50% mass loss at 1 month; > 90% mass loss at 3 months
Akiyama et al. 2024 [25]	BRFD	Rabbit elastase-induced aneurysm	1 month & 3 months (*n* = 4)	1 mo: 2/4 complete occlusion (50%); 3 mo: 4/4 complete occlusion (100%)	No reported analysis	20% mass loss at 1 month; 50% mass loss at 3 months
Oliver et al. 2025 [27]	BRFD	Rabbit elastase-induced aneurysm model	3 months (*n* = 7)	0% complete occlusion (5/7 patent, 1/7 partial, 1/7 parent artery + aneurysm occluded)	One case of parent artery thrombosis	Bioresorbable FeMnN wires degraded too rapidly; fragmented away from aneurysm neck before complete neointimal coverage

*Indicates that the value was estimated using ImageJ to take measurements on published figures. *BRFD* Bioresorbable flow diverter; *NA* Not available, the metric exists for the device but figures that could be used to calculate the metric were not provided in the original manuscript; *FD* Flow diverter; NA, the given metric cannot be measured for the device; (sh), shelved; (unsh), unshelved

### Risk of bias

The SYRCLE-I assessment tool was utilized to assess the quality of the studies through visual representations (Table 1 – Supplementary Material).

## Results

The initial search retrieved 17,883 records from PubMed, Scopus and EMBASE. Before screening, 429 non-English records were excluded. During the screening phase, 15,284 records were excluded for not meeting the inclusion criteria, or for being duplicates. Of the 2170 reports assessed for eligibility, 1784 were excluded due to impertinent or insufficient data, 279 reported bioresorbable devices not used for neuro-endovascular purposes, and 97 because presented insufficient details on device composition and material properties, which limited assessment of bioresorbability. Seven studies were included from the forward search. Ultimately, 17 studies were included in the final analysis (Fig. 1). The quality of these studies was assessed using the SYRCLE-I assessment tool to evaluate the risk of bias (Table 1 – Suppl. Material). The study selection process was documented according to the PRISMA 2020 guidelines, with a flow diagram illustrating the phases of identification, screening, eligibility assessment, and final inclusion (Fig. 1).

### Qualitative analysis (systematic review)

A total of 17 studies published between 2013 and 2025 were included in this systematic review [8, 9, 11, 12, 15–27]. Overall, 27 experimental BRFDs were identified, with wide variability in their materials and design parameters (Table 2). About half of these prototypes (13/27, 48.1%) were fully bioresorbable, consisting entirely of absorbable polymer and/or metal components that can degrade in vivo [11, 12, 16, 17, 21, 22, 25]. The remaining devices were hybrids that included some permanent elements like metal marker wires. Eight devices (29.6%) were reported to be compatible with standard microcatheter delivery [11, 15, 22], whereas eight (29.6%) required balloon assistance for deployment [11, 16, 25, 27]. The need for balloon expansion was noted in certain polymer or magnesium-based designs to ensure proper wall apposition of the FD.

The experimental BRFDs can be categorized as polymeric, metallic, or composite. Fully polymeric scaffolds were common, most often using poly-L-lactic acid (PLLA) fibers or occasionally polycaprolactone (PCL) as the braid material. Several devices employed bioresorbable metal alloys, predominantly magnesium-based alloys, as the primary scaffold wires. Many prototypes were hybrid designs combining polymer and metal. The prevalence of PLLA is notable: this polymer was featured in a 11/27 (40.7%) devices either alone or as the matrix in polymer–metal hybrids [8, 9, 11, 18, 20, 21, 25, 26]. Magnesium was the next most common resorbable material, used in 5 devices as the primary scaffold metal, either with polymer coating or coupled to permanent marker wires [24, 25, 27]. Three (11.1%) designs explored iron-based alloys as bioresorbable metals, typically paired with permanent tantalum marker filaments for visibility [23, 24, 27].

The strut thickness and mesh density varied widely across the 27 BRFDs. Strut diameters ranged from 25 to 50 μm in braided polymer or thin-wire devices up to 350–400 μm in some early polymer prototypes. Most devices achieved porosities in the 60–80% range, comparable to conventional FDs [8, 9, 11, 12, 15–27]. Some outliers were noted: one dense PLLA braid had only 40% porosity, whereas a high-porosity polymer device reached 90% open area [11, 17]. Pore likewise varied, generally falling between single-digits and a few tens [8, 9, 11, 12, 15–27]. Many designs targeted roughly 10–25 pores/mm², although an extremely thick-strut PCL device had < 1 pore/mm² due to its large fiber size [12].

Because fully bioresorbable polymers and magnesium are radiolucent, most BRFDs incorporated some form of radiopaque marker. Embedded metal wires or bands were a common solution: several devices wove platinum or tantalum wires into the braid [18, 21, 26], and others attached gold marker bands at the ends of the stent [11, 21, 25]. In total, 70% of the prototypes included dedicated radiopaque components. An alternative strategy used in polymer-based FDs was to incorporate radiopaque fillers or coatings like barium sulfate (BaSO₄) or bismuth oxide particles blended into a PCL scaffold to make it fluoroscopically visible [12, 22]. A minority of devices had no radiopaque marker and relied on imaging the device via advanced imaging (OCT, micro-CT) rather than standard fluoroscopy [16, 20].

### In vivo performance of bioresorbable flow diverters

Eighteen distinct BRFD devices were tested in vivo (Table 3) [11, 15, 16, 18–21, 25–27]. High aneurysm occlusion rates were achieved in the majority of cases: 11 out of 18 devices (61.1%) induced ≥ 80% aneurysm sac occlusion at one or more follow-up time points [18–21, 25, 26]. Notably, complete aneurysm obliteration by the final follow-up was reported for 6 devices (33.3%) [16, 19, 20, 25, 26]. These successful outcomes were often observed within 3–6 months post-implantation. However, not all prototypes attained high occlusion; several devices demonstrated only partial or no aneurysm thrombosis, underscoring variability in BRFD performance.

In-device thrombosis was relatively infrequent, observed with 4 of 18 devices (22.2%) [9, 11, 15, 27]. Overall, no distal emboli or widespread thromboses were reported in the remaining cases, and any minor mural thrombi were usually confined to areas where struts were malposed. Full device bioresorption was confirmed in approximately half of the tested BRFDs (8/18, 44.4%) by the end of the study follow-up [11, 15, 16, 19, 20, 25–27]. Four (22.2%) devices showed partial degradation over months [20, 21, 25, 26]. Complete or near-complete resorption of the flow diverter was achieved in 4 cases (22.2%) [11, 15, 19, 27]. Only a minority of preclinical bioresorbable flow diverter studies applied standardized clinical angiographic grading systems (Raymond–Roy or O’Kelly–Marotta) [19, 26]. The other investigators assessed aneurysm occlusion using study-specific binary or semi-quantitative angiographic definitions (complete versus incomplete occlusion), reflecting the exploratory and preclinical nature of these studies. Resorption was always assessed using histological analysis.Importantly, even after substantial resorption of polymer or Mg components, any incorporated permanent markers remained visible and no significant chronic inflammation or stenosis was noted in the reported studies.

## Discussion

This systematic review included 17 studies published between 2013 and 2025, describing 27 experimental BRFDs with diverse materials and designs. Across studies, information on deliverability was variably reported, with microcatheter compatibility and balloon use often inconsistently described, while aneurysm occlusion outcomes and resorption profiles showed considerable variability, largely reflecting differences in device composition and structural features.

### Device design

BRFDs have been developed with distinct materials and designs compared to conventional devices like Pipeline, Surpass, and FRED [13] (Table 4). All currently FDA-approved FDs are permanent metal implants made of nitinol or cobalt–chromium alloy, which remain in the artery for the patient’s lifetime [4–8]. The defining feature of BRFDs is that they gradually degrade in vivo, unlike permanent metal implants. This degradation behavior is linked to treatment efficacy and safety. This fundamental difference in composition leads to several notable distinctions in device properties.

**Table 4:** Summary of evidence on bioresorbable flow diverters (BRFDs)

Category	Key findings
Established findings	Feasibility of BRFD deployment in preclinical models; proof-of-concept aneurysm occlusion demonstrated in in vitro and animal studies; predictable material degradation achievable through polymeric or metallic design; acceptable periprocedural safety profiles in controlled experimental settings.
Promising preclinical results	Progressive endothelialization and neointimal coverage observed in selected models; aneurysm occlusion rates comparable to metallic flow diverters at intermediate follow-up in some studies; potential for temporary scaffolding with eventual implant removal through resorption.
Unresolved challenges	Long-term durability of aneurysm occlusion after complete scaffold resorption; optimal matching of degradation kinetics to aneurysm healing biology; risk of late recanalization or delayed thrombosis; variable radial force and time-dependent mechanical support; undefined antiplatelet requirements after partial or complete resorption; lack of human clinical data.

The ideal scenario is that the device remains intact and supports aneurysm occlusion until permanent tissue healing has occurred, then resorbs cleanly without leaving obstructive remnants or causing inflammation. Achieving this ideal is challenging, and different materials have different degradation profiles that impact clinical performance.

BRFDs can be categorized by their primary scaffolding material into polymers, corrodible biomaterials or a combination of both. Other polymers like polycaprolactone have also been explored for their flexibility and slower degradation.

Conventional metallic flow-diverters are braided from high-strength metal wires typically 25–30 μm in diameter. Additionally, FDs must balance porosity to reduce aneurysm inflow without occluding vital branches. In fact, FDA-approved metal-devices have porosities in the 50–70% range (Pipeline 65%; Surpass 70%; FRED 50–60%). In contrast, BRFDs rely on polymeric or biodegradable metallic materials with lower intrinsic strength, making strut thickness and porosity even more critical design parameters. Reported bioabsorbable prototypes employ thicker struts (approximately 41.7 μm for PLLA-based devices and 56.8–74.1 μm for PCL-based devices), which enhances flexibility and conformability and may reduce thrombogenicity, but must preserve adequate radial support and sustained flow diversion. Similar to metallic devices, maintaining porosity below 70% appears necessary for effective flow diversion and intra-aneurysmal thrombus formation, while higher pore density further improves flow disruption at the aneurysm neck and promotes neointimal coverage.

Biodegradable polymers like PLLA typically break down by hydrolysis into lactic acid oligomers over months to years and require thicker struts or higher mesh density to achieve sufficient vessel support. For example, Tidwell et al. employed a stent 350 μm in diameter, roughly 10 folds thicker than the metal wires in the pipeline FD (25–30 μm in diameter) [12]. Even the other PLLA BRFDs analyzed had a diameter ranging between 30 and 74 μm, still exceeding typical metal strut sizes [8, 11, 18, 20]. As expected, thicker struts are generally less flexible, thus limiting their deliverability: in fact, some devices couldn’t be deployed through standard microcatheters and required surgical implantation in mice models. Some designs initially required modifications: for example, a bioresorbable magnesium alloy stent in a rat model needed balloon angioplasty to ensure it opened fully [11, 15]. Indeed, some second-generation polymer-based BRFDs have been designed to fit 0.027–0.033″ microcatheters [19, 26].

Radiolucency on fluoroscopic imaging remains a great challenge that has yet to be solved. Metals like platinum or tantalum are integrated into standard FDs to enhance visibility under fluoroscopy: in particular Pipeline incorporates 12 platinum wires, while FRED has tantalum markers.

Newer bioresorbable devices have started weaving a small number of platinum or tantalum wires into the braids [8, 18, 19, 26], adding gold markers at device ends [11, 21, 25] or coating fibers with radio-opaque particles like BaSO₄, or Bi₂O₃ to allow for better visualization during deployment [12, 22]. As a result, these devices are not fully bioresorbable as the metal remnants persist. However, a benefit of reduced metallic content is improved imaging post-procedure: one study noted a progressive loss of radiopacity on CT and only mild MRI artifacts as a polymer stent gradually resorbed over 6 months [20]. This suggests follow-up imaging of treated aneurysms might be easier with BRFDs, due to minimal long-term artifact once the device dissolves.

Differently, metal-based BRFDs are generally made of biocorrodible alloys like iron or magnesium. The metal wires can be made very thin yet still pertain high elastic and tensile strength. Magnesium- and iron-based prototypes used wire diameters of 46–65 μm [25] and 25 μm [23, 27] respectively, similar to current permanent FDs. A great advantage of metal devices is their inherently radiopacity with some degree of variation: iron is more visible than magnesium on fluoroscopic imaging. Nevertheless, currently developed metal-devices still employ a small amount of tantalum or platinum to enhance visualization on imaging [27].

Both polymer and metal BRFDs report porosity in the 60–80% range and pore density in the 5–20 pores/mm² to balance flow diversion/perfusion rates. For instance, a PLLA BRFD had 60% porosity with 20 pores/mm^2^, comparable to a metal FD’s 13–14 pores/mm^2^ [21]. Some early prototypes explored higher porosity (90%) to preserve branch flow, but very high porosity can sacrifice flow-diversion efficacy [17]. However, obtaining these parameters often requires a higher filament count or braid angles. For example, the PLLA device by Nishi et al. achieved 60% porosity using 48 fibers at 40° braid angle, while metal resorbable devices can generally reach similar porosity with fewer wires thanks to their thinner struts [11].

Future studies involving human subjects will be essential to determine the clinical applicability of bioresorbable FDs, particularly in light of the substantial heterogeneity in device design, materials, degradation profiles, experimental models, follow-up durations, and outcome measures across existing preclinical studies, which currently limits robust cross-study comparison and generalization of efficacy and safety findings.

### Biocompatibility, and degradation profiles

In the intracranial context, a relatively slow degradation is desirable so that the scaffold persists through the critical healing window [8, 11, 18, 20]. The PLLA FD studied by Nishi et al. lost only 15% of its molecular weight in 3 months, 45% by 9 months, and almost entirely degraded by 1.5 years [11]. Similarly, Muram et al. and Morrish et al. both found complete resorption of the PLLA stents by 1 year. Sasaki et al., reported that their PLLA stent exhibited 50% mass loss by 12 months in rabbits, with scanning electron microscopy confirming progressive thinning of the fibers over time. The degradation of PLLA was associated with a moderate transient inflammation, but importantly no deleterious vessel reactions were seen long-term: by 12 months the inflammatory response subsided, and neointimal tissue in the BRFD-treated arteries was qualitatively healthy, with collagen-rich matrix similar to normal healing [21]. This indicates that the biocompatibility of PLLA is acceptable, in line with its long history of safe use in biodegradable sutures and coronary stents [8, 11, 18, 20]. One safety consideration is the production of lactic acid as a breakdown product, which can transiently lower local ph. However, in cerebrovascular implants the small mass of PLLA used is unlikely to cause any systemic effect; local tissue can buffer these byproducts, and the gradual pace of degradation allows for clearance. Indeed, none of the animal studies reported aneurysm wall inflammation or degeneration beyond what is expected during normal aneurysm healing [8, 11, 18, 20].

Faster resorbing polymers like polyglycolic acid (PGA) have been tested in early prototypes: Wang et al. braided PGA fibers with metal wires and observed that PGA struts were already mostly resorbed by 6 weeks, completely disappearing by 3 months. Such rapid loss of support, however, risks premature loss of flow diversion. PLLA’s longer persistence is generally preferred for durable aneurysm occlusion.

Instead, bioresorbable metal-based devices undergo corrosion in vivo, transforming into oxides or salts that can be absorbed or excreted by the body. Magnesium alloys typically corrode faster than iron alloys. Rapid corrosion can be a double-edged sword: it ensures the implant doesn’t linger too long, but if too rapid, it can result in premature loss of scaffolding and even gas release. Gas pockets from hydrogen evolution typically dissolve or are small enough to be benign to determine adverse effects. In vascular settings, magnesium stents tended to lose mechanical integrity within 2–3 months, which in coronaries led to higher risk of early recoil and thrombosis in first-generation devices [28]. Akiyama et al. (2025) directly addressed magnesium’s fast corrosion by adding a PLLA polymer coating to their braided magnesium FD [25]. This thin polymer coating acted as a barrier to slow corrosion, effectively “dialing down” the resorption rate. Their results demonstrated that uncoated Mg stents lost 50% mass by 1 month and > 90% by 3 months, whereas PLLA-coated Mg stents lost only 20% by 1 month and 50% by 3 months [25]. The coated magnesium device thus maintained its structural integrity far longer. The authors concluded that the PLLA-coated Mg BRFD had greater clinical feasibility due to its delayed bioresorption [25]. Importantly, the coating did not provoke any new inflammation, and the vessel healing was similar between the two, aside from the difference in how long struts remained visible on imaging.

Iron-based BRFDs generally corrode more slowly than magnesium. Pure iron implants in arteries have been shown to persist for many months, sometimes too long to be practical. Alloying iron with more reactive elements such as manganese, zinc, or creating Fe-based composites, is one way to accelerate bioresorption. Oliver et al.’s FeMnN alloy (35% Mn, 0.15% N) was an attempt to create an iron-based wire that would resorb in under a year [24, 27]. At 3 months, the braided FeMnN device had partially fragmented, with many wire segments completely dissolved or dislodged from the aneurysm neck region. Interestingly, in cases where iron wires remained in place, histology showed robust tissue incorporation. The rapid degradation was implicated in the failure to occlude aneurysms. Research is ongoing into novel alloys to achieve a balanced corrosion rate. For example, other studies have evaluated iron stents with alloying elements like molybdenum or phosphorus to fine-tune the resorption to an acceptable timeline [27].

In summary, both polymer and metal BRFDs can be engineered to degrade safely, without imposing an excessive thrombotic burden, but the window of resorption must be matched to the biology of aneurysm healing. Too rapid resorption can lead to recanalization, while overly prolonged resorption blunts the benefit of removing the implant. These considerations are currently supported by in vitro and non-human studies and should therefore be interpreted as hypothesis-generating rather than indicative of clinical performance in humans, particularly given the substantial heterogeneity in device designs, materials, degradation kinetics, experimental models, follow-up durations, and reported endpoints across the available literature.

### Mechanical and flow-diversion performance

A critical question for BRFDs is whether they can match the aneurysm occlusion efficacy of permanent devices during the period before they dissolve. Across multiple preclinical studies, BRFDs have shown promising occlusion rates that are broadly comparable to conventional FDs, though results vary with device generation and study design. Overall, the evidence suggests that if a BRFD provides sufficient flow diversion for the necessary duration, aneurysm thrombosis and healing can proceed effectively.

Early proof-of-concept came from Wang et al. (2013), who implanted a PGA BRFDs in rabbit aneurysms [15]. At 3 months, complete aneurysm occlusion was achieved in 83.3% of cases treated with the PGA-based BRFD, whereas 0% of aneurysms treated with a comparable permanent metal stent were occluded in that timeframe [15]. This stark contrast (83% vs. 0% occlusion at 3 months) demonstrated the flow-diverting capability of the biodegradable device [15]. Notably, no side branch occlusion occurred despite one group of rabbits having the BRFD covering a major branch artery. This finding supports a theoretical advantage of BRFDs: they provide temporary flow diversion to induce aneurysm healing, but ultimately spare jailed branch vessels from permanent coverage. Branches remained patent once the device resorbed, with neointimal tissue sealing the aneurysm neck only.

Subsequent studies have refined BRFD designs and assessed outcomes at longer follow-ups. Nishi et al. (2019) tested a fully bioresorbable PLLA FDin elastase-induced rabbit aneurysms over 1 year. Occlusion developed more gradually in this model: 0% at 1 month, 20% at 3 months, and 50% by 6 months, reaching 67% (2 of 3 aneurysms) by 12 months [11]. While not all aneurysms were occluded at one year in that pilot study, the authors noted that no parent artery thrombosis or side branch occlusion occurred, and any thrombus formation on the device was limited to small, resolving deposits seen on CT imaging. The need for longer follow-up was emphasized, as PLLA is relatively slow to resorb; indeed, in vitro degradation tests showed the device retained 55% of its molecular weight at 1 year [11].

More recent second-generation BRFDs have demonstrated faster and more consistent aneurysm healing, in part by optimizing the balance between flow diversion and degradation time. Sasaki et al. (2023) directly compared a 48-strand PLLA FD to a standard cobalt–chromium device in identical rabbit aneurysm models [21]. By 3 months post-treatment, 73% of aneurysms treated with the PLLA BRFD showed Raymond grade I or II, indicative of substantial occlusion, which was similar to the 80% occlusion rate with the metal stent. By 12 months, 100% of aneurysms in both groups were occluded [21]. There was no significant difference in the proportion of aneurysms achieving neck remnant or complete occlusion between the bioresorbable and permanent stent groups [21]. Interestingly, the PLLA group had a higher rate of complete occlusion (48% vs. 13% in the metal group at one year), suggesting the possibility that a resorbable scaffold might even accelerate complete healing in some cases. Importantly, no in-stent thrombosis or branch occlusions were observed in either group, indicating that the BRFD’s performance in vivo was comparable with the clinically proven metallic device in terms of safety. Histopathology in this study confirmed similar neointimal formation at 12 months for both devices, with the PLLA stent causing a transient inflammatory response but ultimately yielding a stable healed aneurysm neck indistinguishable from that of the metal stent. This head-to-head comparison provides strong evidence that BRFDs can be as effective as cobalt–chromium devices in achieving aneurysm occlusion over 1 year [21].

Another encouraging example comes from the ReSolv™ BRFD stenting [26]. In a rabbit saccular aneurysm study by Morrish et al. (2024), the ReSolv device showed 85.7% adequate occlusion by 7.5 months follow-up, with complete aneurysm occlusion in 64.3% of cases [26]. All parent vessels (14/14) and all jailed side branches (33/33) remained patent at last follow-up. No significant in-stent stenosis or thrombosis was seen, and angiographic apposition was good in all treated arteries. By 6 months, a high proportion of ReSolv-treated aneurysms had progressed to complete occlusion, and by 9–12 months essentially all were cured [20]. These results mirror the performance of traditional FDs in similar rabbit models, which typically achieve 75–100% occlusion by 6–12 months depending on device porosity [29]. Notably, the polymer scaffold of the ReSolv stent was partially resorbed by 6 months, and ongoing studies aim to observe the outcomes after full absorption of the polymer component. The fact that aneurysm occlusion was obtained before the device disappeared in these animals is a positive sign for the concept of “treat and vanish”. If an aneurysm has not completely healed when the device starts to weaken, there is a risk of recanalization or device migration. Nevertheless, given the short follow-up protocols of the included studies, the long-term durability of aneurysm occlusion after full scaffold resorption remains unknown, and the possibility of late recanalization cannot currently be excluded.

It is also informative to consider cases where BRFD performance has been suboptimal, as these highlight the importance of matching device persistence to biological healing. Oliver et al. (2025) evaluated a novel iron-based alloy (FeMnN) BRFD in rabbit aneurysms, which unfortunately failed to occlude aneurysms in 5 out of 7 cases by 3 months, with 0% complete occlusion achieved [27]. In fact, most aneurysms remained fully patent at 3 months and one case suffered thrombosis of the parent artery along with the aneurysm. Premature or excessively rapid device resorption, due to a mismatch between degradation kinetics and the biological timeline of aneurysm healing, represents a critical potential failure mode for bioresorbable FDs and underscores an unresolved safety concern that warrants cautious interpretation of the current preclinical data. The investigators found that the FeMnN alloy wires degraded too rapidly, fragmenting and losing structural integrity at the neck before a stable neointimal seal could form [27]. Essentially, the scaffold vanished in a matter of weeks, leading to persistent aneurysm flow and increasing the risk of device fragment embolization, termed the “harpoon” effect in their report [27]. This outcome underscores a key lesson: premature resorption can undermine treatment efficacy [13]. A FD must maintain coverage of the aneurysm neck for a sufficient period to allow endothelium and collagen to permanently wall off the aneurysm. If the device resorbs faster than the healing process, aneurysm recanalization or treatment failure may result. In our analysis, 11 out of 18 devices (61.1%) achieved ≥ 80% aneurysm sac occlusion, while complete obliteration at final follow-up was reported only for 6 devices (33.3%). While encouraging within preclinical contexts, occlusion rates remain lower than rates typically reported for conventional permanent flow diverters in clinical practice (80–86%).

Additionally, all studies employed DAPT during the implantation period, and as a result acute thrombosis on the devices was minimal. Of note, our analysis showed thrombosis only in 4/18 devices [11, 15, 19, 27]. Small thrombi were occasionally seen adherent to struts by scanning electron microscopy or OCT imaging [9], but these did not lead to vessel occlusion or clinical stroke in the animal models. Collectively, these findings indicate that BRFDs can be deployed with similar periprocedural safety profiles to standard FDs, at least in the controlled setting of animal studies. However, this potential advantage remains theoretical, as all available preclinical studies employed dual antiplatelet regimens with variable protocols and durations, and antiplatelet requirements after partial or complete device resorption are currently undefined. The combination of effective aneurysm occlusion preserved branch flow, and eventual implant elimination positions BRFDs as a very attractive next-generation solution, provided these outcomes translate to human anatomy and longer time scales.

### Translational gap

Despite promising results in preclinical models, a clear translational gap persists between BRFD development and clinical application. Most evidence derives from benchtop and animal studies that do not fully capture the anatomical, hemodynamic, and long-term biological complexity of human intracranial aneurysms. Moreover, heterogeneity in device design and outcome definitions limits direct extrapolation to clinical practice. As such, while BRFDs represent an attractive next-generation concept, their safety, durability, and efficacy in humans remain to be established in well-designed clinical studies.

### Limitations

Despite all the headways we’ve made in testing FDs there are still some gaps to be filled. All of what we know far comes from testing animals and in labs. Several included studies are proof-of-concept experiments presenting early-stage prototypes that are not intended for human use. Not a single BRFD has been approved for use in people, and no clinical trials have been published on the subject. Additionally, each study uses different protocols and in vivo models for testing: some rely on rabbit elastase induced aneurysms, while others on swine or canine models. However, each of these models has its unique flow dynamics and ways of healing which may confound the interpretation of results. The immunological and molecular mechanisms underlying inflammation and vascular healing after BRFD implantation remain largely unexplored, as no available studies have directly investigated these processes, and any further mechanistic discussion would therefore be speculative.

Furthermore, the included articles look at different outcomes: some deal with biomechanical dynamics while others are focused on tissue growth or changes seen on images. The time between follow ups can be anywhere from a week to over a year which makes it difficult to compare results directly. Additionally, current follow-ups should be extended, as they cannot predict the long-term changes of patients with a BRFD-treated aneurysm. Longer observation is necessary to clarify the recurrence rate of aneurysms after device resorption, to understand the histopathological changes of the vessel wall over time, and to identify potential late-onset adverse effects.

The included studies fail to report on the length of time for using DAPT for dissolvable stents. Most stent patients are required to take aspirin for the rest of their lives and Plavix for a few months. With the dissolvable kind, which typically disappears after about a year, it’s possible that patients will not need any of these medications once the stent is fully resorbed. In the included studies, authors administered a DAPT regimen only for a short time after delivering the implant. However, it might be that patients who get these implants may need to stay on blood thinners until the device is fully broken down by the body to prevent any blood clots from forming. However, if these devices prove effective, they could reduce the duration of DAPT required, which would represent a significant advantage for patients that can’t adhere to the DAPT for too long due to the underlying comorbidities. Thus, while the current evidence is encouraging, it remains preliminary and should be interpreted as preliminary.

## Conclusion

BRFDs are advancing rapidly through innovations in materials, braiding, and coating strategies aimed at replicating or improving the performance of metallic devices. Further validation in large-animal models and early clinical trials will be essential to establish safety, efficacy, durability, and optimal antiplatelet regimens. With successful translation, BRFDs could expand to complex aneurysm treatment and offer the distinctive benefit of aneurysm cure without a permanent implant. Yet, those results remain purely speculative and preclinical.

## Supplementary Information

Below is the link to the electronic supplementary material.

Supplementary Material 1 (XLSX 9.23 KB)

## Data Availability

No datasets were generated or analysed during the current study.
